# Supplementary material for: Patterns of Intron Gain and Loss in Fungi
Source: PLoS Biol. 2004 Nov 30;2(12):e422. doi: 10.1371/journal.pbio.0020422 (PMC532390; doi:10.1371/journal.pbio.0020422)
Supplement: Table S1 — Also available at http://genes.mit.edu/NielsenEtAl/. (4.3 MB ZIP). [file pbio.0020422.st001.zip › NielsenEtAl/html/445.html]

AN3829.1.NCU00936.1.MG01230.1.FG06752.1


```
 CLUSTAL W (1.82) Multiple Sequence Alignments - Introns Inserted


Sequence 1: AN3829.1	524 aa
Sequence 2: NCU00936.1	494 aa
Sequence 3: MG01230.1	536 aa
Sequence 4: FG06752.1	490 aa
Alignment Length: 551 aa
Number Identitical Residues: 262 aa
Alignment Score (without introns) 12712


MG01230.1 	MSAIRMHRNACSASRLVARTSAPRRTVPFLSPSYARLASTQVPK0LKDPSLFKQNVCYVN
NCU00936.1	----------------------------------------MAPK0LKDPSLFKKDVCYVN
FG06752.1 	---------------------------------------MASPK0LSDPSLIKNA-CYVN
AN3829.1  	-------------------------MPSHTYPQRLYTTGYTVPP0LKDKSLFIQK-AFVN
          	                           .   ..    :.   *  *.* **: :  .:**

MG01230.1 	GAFVPAKSGATFAVA-~-------------DPT~TGEHIGDAPEFDAADTEAAIAAAENA
NCU00936.1	GEWVKAHSGKTFEVN-~---------------A1TGKLIGTCPEFDAQDTKKAIDAAETA
FG06752.1 	GQWVAAKSGKDFSVEN1--------------PA~SLEKLGSCPEFDANDTEAAIAAADAA
AN3829.1  	GEWVDAESGKTFEVHA1HIDANGWYLDVLTDPA~TGKLIGTCPEFSASDTEKAIQAASAA
          	* :* *.**  * *     .:..   .  :..: : : :* .***.* **: ** **. *

MG01230.1 	FKTYRLTTGRERSKLLRRWYDLMIANADDIATLITWENGKTIADAKGEVTYAANFFEWFS
NCU00936.1	FETFRHKTGRERSKLLRKWYDLMVENHDDLTTLITLENGKPLADAKGEVTYAANFFEWFS
FG06752.1 	YKTYRKTPARQRARYLRRWYDLMMENADDIARLITLENGKVWSDAKTEAVYAANFFEWFS
AN3829.1  	FPKFRATLARERARMLRRWYQLMVDNAEDLATLITWENGKPLADAKGEVNYAASFFEWFS
          	: .:* . .*:*:: **:**:**: * :*:: *** ****  :*** *. ***.******

MG01230.1 	EEAPRVYGDTIQPTLAANRVVTRKEPVGVCSLITP2WNFPAAMGTRKIGPALAAGCTVVC
NCU00936.1	EEAPRIYGDTIPSSVPGNRVWTIKEPVGVCGLITP~WNFPAAMITRKVGPALAVGCTVVC
FG06752.1 	EEAPRIYGETIEASNPSCRLSTIKQPVGVCGLIAP~WNFPAAMITRKAGPALAAGCTVVI
AN3829.1  	EEAPRTYGDTIPASVPGNRVITVKQPVGVCGLITP~WNFPAAMITRKIGPALAAGCTVVT
          	***** **:** .: .. *: * *:*****.**:* ******* *** *****.***** 

MG01230.1 	KAPAETPFTSLALAELAHRAGVPAGVVNFVTSHSNTKTVGEVLCTHQAVRKISFTGSTGV
NCU00936.1	KAPGETPFTALAIAELAHRAGIPPGVVNVITALENTPEVGSTLTTDPVVRKISFTGSTAV
FG06752.1 	KAPAEAPLTALALAELAHRAGIPAGVVNIITALDNTAEVGKVLTTHPKIKKVSFTGSTGV
AN3829.1  	KSPGETPFTANALAELANRAGIPKGVVNIVTAMKNTPEVGEMITTHPDIRKVSFTGSTNV
          	*:*.*:*:*: *:****:***:* ****.:*: .**  **. : *.  ::*:****** *

MG01230.1 	GKTLMQQAAGTLKKCSFELGGNAPFIVFDD-ADLDAAVDGAIACKFRSSGQTCVCANRIY
NCU00936.1	GKLLMKQCSGTLKKLSLELGGNAPFIVFDD-ADVDAAVTGAIASKFRSSGQTCVCANRIY
FG06752.1 	GRLLMNQSSSTVKKLSFELGGNAPFIVFED-ADLEKAVKGIITSKFRNSGQTCVCANRIF
AN3829.1  	GRLLMKQSSSTIKKVSWELGGNAPFIVFDDVEDLDAAVTGAIASKFRSSGQTCVCANRIY
          	*: **:*.:.*:** * ***********:*  *:: ** * *:.***.***********:

MG01230.1 	VQRGIYDAFAERLAKRVKETFKIGSGFDPETTHGPLIHDRAVSKVASQVEDAVGKGAKLL
NCU00936.1	VQRGIYDEFSQKFAEQVKNNFRVGNGFEEGVTHGPLIHHRAIEKVEQHVRDAEKKGAKVV
FG06752.1 	VHRSIYDKFVQMVLDVVKT-FVIGDGFGEKTTHGPLIHGRAVAKTAEHVEDAISKGAKLI
AN3829.1  	VQKGIYDEFVQKFVEKVRN-FKVGAGFEDGVTHGPVIHDRAVDKVDQHVQDAISKGAKLI
          	*::.*** * : . . *:  * :* **   .****:** **: *. .:*.**  ****::

MG01230.1 	AGGKRLPDLGPHFFAPTVLGDMTPEMSIAREETFGPIAGLFKFDTEAEVVKMANDTEVGL
NCU00936.1	VGGHRLESLGPNFYEPTVITGMTPDMAMASEETFGPVAGLFPFDTEDEVVKLANATQVGL
FG06752.1 	HGGERLADLGPNFFGLTMLTDMTPDMKIFSEETFGPVAAFFAFDTEEEVIELANDSEVGL
AN3829.1  	AGGQRRSDLGPNFYDLTVLANMTKDMKIASEETFGPVAGLFPFETEKEVVELANKAEVGL
          	 **.*  .***:*:  *:: .** :* :  ******:*.:* *:** **:::** ::***

MG01230.1 	AGYFFSKDIHRAYRVAEALEVGMVGVNTG~IISDVASP~FGGVKESGFGREGSMYGIHEY
NCU00936.1	AGYFFSRDIHRCVRVAEHLEVGMVGINTG~LISDPASP~FGGVKESGFGREGSLYGIGEY
FG06752.1 	GGYFFSENVNRCYRVAEALEVGMIGVNCG1VLSDPAAP~FGGIKQSGFGREGSKYGIDEF
AN3829.1  	AGYFFSGNIKRIFRVAEALEVGMVGVNTG~LISDVASP2FGGVKQSGFGREGSKYGIEEF
          	.***** :::*  **** *****:*:* * ::** *:* ***:*:******** *** *:

MG01230.1 	QVTKTITFGGMGKELQG
NCU00936.1	QVTKMVTVGGMGQPLQK
FG06752.1 	TVTKMVMTN-----IDP
AN3829.1  	MTIKSVTFGGMGEPLQS
          	 . * :  .. .. ::
```
